# Prediction of Nocturnal Hypoglycemia Following Exercise in Type 1 Diabetes Using Temporally Structured CGM-Derived Digital Biomarkers

**DOI:** 10.3390/s26123842

**Published:** 2026-06-17

**Authors:** Agnese Piersanti, Gaia Maria Manes, Libera Lucia Del Giudice, Laura Burattini, Christian Göbl, Andrea Tura, Micaela Morettini

**Affiliations:** 1 Department of Information Engineering, Università Politecnica delle Marche, Via Brecce Bianche 12, 60131 Ancona, Italy; a.piersanti@staff.univpm.it (A.P.); s1120419@studenti.univpm.it (G.M.M.); l.l.delgiudice@pm.univpm.it (L.L.D.G.); l.burattini@staff.univpm.it (L.B.); 2 Department of Obstetrics and Gynaecology, Medical University of Vienna, Waehringer Guertel 18-20, 1090 Vienna, Austria; christian.goebl@meduniwien.ac.at; 3 CNR Institute of Neuroscience, Corso Stati Uniti 4, 35127 Padova, Italy; andrea.tura@cnr.it

**Keywords:** nocturnal hypoglycemia, type 1 diabetes, continuous glucose monitoring, exercise, machine learning, SMOTE, feature engineering

## Abstract

Nocturnal hypoglycemia (NH) following exercise represents a critical challenge in the management of type 1 diabetes (T1D), particularly in pediatric populations, where its occurrence is associated with severe adverse outcomes and increased caregiver burden. This study aimed to identify an interpretable early signature based on CGM-derived digital biomarkers of post-exercise NH risk in children and adolescents with T1D. CGM data from 49 pediatric subjects (DirecNet cohort) were used to extract several CGM metrics across two temporal configurations: (i) Exercise + Cumulative, where features were computed over the exercise window and over an extended window spanning from exercise onset through recovery (16:00–17:00 and 16:00–22:00); and (ii) Exercise + Post-exercise, where features were computed separately over two non-overlapping intervals, capturing the exercise phase and the subsequent recovery phase (16:00–17:00 and 17:00–22:00). A Random Forest classifier was trained within a Leave-One-Out Cross Validation framework, incorporating variance inflation factor (VIF)-based multicollinearity filtering, minimum redundancy–maximum relevance (mRMR) feature selection, and SMOTE-based class balancing. The Exercise + Post-exercise configuration achieved superior performance: balanced accuracy (BA) = 76.9%, F1-score = 0.71, Area Under Receiver Operating Characteristic Curve (ROC-AUC) = 0.75, outperforming the Exercise + Cumulative configuration; this result was achieved using only five features: CONGA-15_EX (short-term glucose variability during exercise) emerged as the most robust predictor, alongside below_54 and above_250 (time spent in hypoglycemic and hyperglycemic ranges), MAG (mean absolute glucose change), and GRADE_hypo (hypoglycemia risk score). The generalizability of the temporal framework was further supported by independent validation on the OhioT1DM free-living cohort, where the Exercise + Post-exercise configuration (BA = 76.3%, ROC-AUC = 0.804) again outperformed the cumulative approach. These results suggest that a small set of interpretable CGM-derived features, extracted from the exercise and recovery windows, can effectively discriminate pediatric T1D subjects at risk of NH, supporting the development of lightweight CGM-only decision support tools for safer exercise management.

## 1. Introduction

Hypoglycemia, defined as a drop in blood glucose levels, represents the most critical challenge in the clinical management of patients with type 1 diabetes (T1D) [1]. Physical exercise may act as a trigger for hypoglycemia, which may occur not only during exercise, but also within a 6–15 h window following an exercise session in the immediate recovery phase, or even overnight [2]. This risk is further amplified in the pediatric population, since the glycemic response to exercise is particularly unpredictable owing to fluctuating growth hormone levels and the variable intensity of spontaneous physical activity [1]. Data from the Diabetes Control and Complications Trial (DCCT) revealed that 43% of all hypoglycemic episodes and 55% of severe episodes in T1D occur at night, with 51% of severe cases going undetected during sleep. While day-time episodes can lead to dangerous situations like falls or accidents, severe nocturnal hypoglycemia (NH) is particularly hazardous, potentially leading to seizures or “dead-in-bed syndrome”, a phenomenon estimated to account for 5–6% of deaths in T1D patients under 40 years of age. In the pediatric population, NH is of particular clinical concern, as it may impair cognitive function and academic performance while substantially increasing caregiver burden, frequently necessitating repeated nocturnal glucose monitoring by parents or caregivers [3]. As a result, children and adolescents with T1D are often discouraged from engaging in regular physical activity, although it is strongly recommended as it improves cardiovascular health and insulin sensitivity [2,4,5].

In this context, preventive actions to reduce adverse glycemic events are crucial and continuous glucose monitoring (CGM) systems may act as an important source of information. By frequently measuring interstitial glucose, CGM systems already provide prediction on the occurrence of specific glycemic events. However, prediction of exercise-related hypoglycemia remains challenging. The increasing availability of CGM data, together with advances in machine learning (ML), has therefore stimulated growing interest in data-driven approaches for hypoglycemia prediction and risk stratification. ML approaches for hypoglycemia prediction in T1D span two paradigms: glucose forecasting over short horizons (typically 30–60 min) and event classification predicting the binary occurrence of future hypoglycemia, as recently reviewed in [6]. While forecasting models have achieved strong performance in short-term prediction, event classification approaches are more directly suited to anticipatory risk stratification, which coincides with the scenario addressed in the present work. At the longer end of the classification spectrum, Cichosz et al. developed a model predicting weekly hypoglycemia risk from CGM-derived metrics, demonstrating that CGM features encode substantial information about future risk even at the weekly timescale [7].

Several studies have also investigated the prediction of exercise-induced hypoglycemia. Turksoy et al. proposed an approach based on energy expenditure and exercise type [8], while Reddy et al. employed Decision Tree and Random Forest models, identifying baseline glucose levels less than 180 mg/dL and heart rate higher than 120 beats per minute as key predictors of hypoglycemia during physical activity [2,9]. Prasanna et al. [10] identified lower pre-exercise glucose levels and the 2-h pre-exercise area under the curve (AUC) as a strong predictor of intra-exercise hypoglycemia. Similarly, Bergford et al. [5] emphasized the pre-exercise glucose rate of change as the most important predictor of hypoglycemia during physical activity. More recently, Russon et al. developed GlucoseGo, achieving ROC-AUC of 0.87 using only pre-exercise glucose, exercise duration, and rate of change [11]. In parallel, considerable attention has been devoted to nocturnal hypoglycemia (NH). Herrero et al. demonstrated the feasibility of ML-based NH prediction [12]. A recent study evaluated 14 time-series classification models for NH prediction, finding interval- and feature-based approaches most effective [13]. Comparatively fewer studies have investigated NH specifically in the context of physical activity: Leutheuser et al. [14] and Betarchi et al. [15] developed predictive models integrating both CGM data and wearable-derived physical activity metrics. Of note, progress in this area has also been constrained by limited data availability: as highlighted by Del Giudice et al. in a recent scoping review [16], open CGM datasets suitable for ML model development remain scarce, and recent efforts are moving in this direction although datasets with labeled exercise sessions remain limited [17].

Despite these advances, several research gaps remain. First, many approaches rely on multiple sensing modalities, including wearable-derived physical activity metrics, thereby increasing patient burden and limiting practical deployment. Second, numerous studies utilize raw time-series inputs or automatically learned representations, providing limited insight into the physiological mechanisms associated with hypoglycemia onset. Third, when handcrafted features are employed, they are often extracted from fixed or arbitrary temporal windows rather than from physiologically meaningful periods linked to exercise-related glycemic responses. Finally, most studies focus on general hypoglycemia prediction or NH prediction independently, while comparatively little attention has been devoted to identifying the specific CGM-derived patterns that characterize NH occurring after physical activity. To address these gaps, the present study investigates the prediction of exercise-related NH using exclusively CGM-derived information. To our knowledge, no existing study has systematically examined CGM features extracted from physiologically motivated temporal windows that explicitly separate the exercise phase from the subsequent recovery period for post-exercise NH prediction in pediatric T1D. Rather than relying on multimodal sensing or features extracted from arbitrary time intervals, we derive CGM metrics from physiologically motivated windows surrounding exercise, thereby prioritizing interpretability. The main contributions of this work are: (i) a systematic comparison of two temporal CGM feature configurations demonstrating that explicitly separating exercise and recovery phases yields superior predictive information for delayed NH risk; (ii) an interpretable prediction pipeline that exploits CGM time-series data only, avoiding the need for additional wearable sensors and enabling lightweight integration into existing CGM-based decision support systems; (iii) independent validation on the OhioT1DM free-living cohort confirming the generalizability of the proposed temporal framework; and (iv) to our knowledge, the first systematic investigation of CGM-derived post-exercise NH signatures in a pediatric T1D population.

## 2. Materials and Methods

### 2.1. Dataset Description

The data used in this study were obtained from the publicly available Diabetes Research in Children Network (DirecNet) database [18] and included 50 pediatric subjects with T1D (mean age 14.8 ± 1.7 years; 28 males and 22 females; mean HbA1c 7.8 ± 0.8% (mean ± standard deviation)). The subjects underwent a standardized 24 h inpatient monitoring protocol, including a supervised treadmill exercise session in the late afternoon. Exercise intensity was individually calibrated in the morning to achieve a target heart rate of 140 bpm (55% of maximal effort, i.e., VO2max). Interstitial glucose was recorded every 5 min using a Medtronic MiniMed CGM system (Medtronic MiniMed Inc., Northridge, CA, USA) [19].

CGM recordings were preprocessed according to the DirecNet protocol by defining three standardized time windows for each recording session: the exercise window (16:00–17:00), the post-exercise window (17:00–22:00), and the nocturnal window (22:00–07:00). Each recording session corresponds to a single subject undergoing one monitored exercise protocol. The prediction task was defined as the identification of nocturnal hypoglycemic events using CGM data collected prior to the nocturnal period. Accordingly, each recording session was labeled based on the occurrence of at least one hypoglycemic event during the nocturnal window, defined as interstitial glucose values below 60 mg/dL. This threshold was retained in accordance with the study protocol, as all clinical interventions were based on this definition.

### 2.2. Feature Engineering

The dataset was constructed using a comprehensive set of CGM-derived metrics. A total of 49 CGM metrics were computed using the open-source R package iglu v3.4.2 [20], complemented by custom-developed R functions. The reliability of the iglu-derived metrics was established following the methodology of the study by Piersanti et al., which provides a rigorous evaluation of software packages and tools for the analysis of CGM data [21].

Features were extracted from CGM data collected during the exercise and post-exercise periods and used as predictors of hypoglycemic events occurring in the subsequent nocturnal window. Two feature configurations were considered. In the Exercise + Cumulative configuration, features were computed from the exercise window (16:00–17:00) and from the cumulative window spanning 16:00–22:00. In the Exercise + Post-exercise configuration, features were computed separately from the exercise window (16:00–17:00) and the post-exercise window (17:00–22:00).

**Figure 1:**
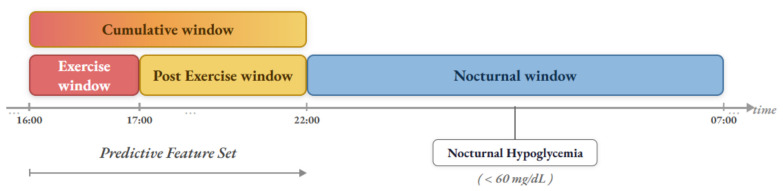
Time-window segmentation for CGM feature extraction and outcome definition.

The computed CGM metrics were selected to characterize glycemic behavior across multiple domains, including descriptive statistics, time-in-range, glycemic risk, glycemic control, and glycemic variability. To specifically characterize acute glycemic responses during physical activity, five additional indicators were computed exclusively within the exercise window (16:00–17:00): mean glucose, standard deviation (SD), coefficient of variation (%CV), mean glucose slope, and the Continuous Overall Net Glycemic Action at 15 min (CONGA-15). These exercise-specific metrics were included to capture the short-term glucose dynamics potentially associated with subsequent NH risk. The complete list of computed metrics is reported in Table S1 in the Supplementary Materials. All metrics were computed using default settings unless otherwise specified, and those requiring a minimum of two days of recording were excluded to ensure consistency. The temporal framework used for CGM feature extraction and NH outcome assessment is shown in Figure 1.

### 2.3. Classification Task

The predictive modeling task was formulated as a binary classification problem at the level of the recording sessions. Each session was classified according to the presence or absence of at least one nocturnal hypoglycemic event (glucose < 60 mg/dL). Features extracted from the exercise and post-exercise periods were used as predictors of this outcome.

### 2.4. Model Framework

**Figure 2:**
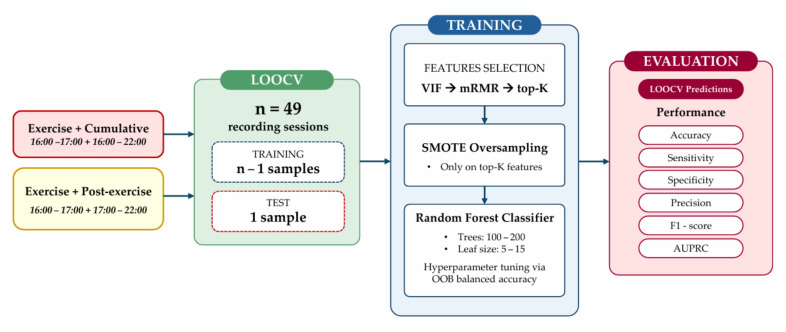
A schematic representation of the modeling framework applied independently to the two temporal analyses (Exercise + Cumulative configuration and Exercise + Post-exercise configuration). For each analysis, zero-variance features were first removed, followed by iterative VIF-based backward elimination to address multicollinearity. Feature selection was performed using the mRMR criterion, retaining the top K features on the real training data. Class imbalance was corrected using the Synthetic Minority Oversampling Technique (SMOTE, with number of nearest neighbors = 5). A Random Forest classifier was trained with hyperparameter tuning guided by Out-Of-Bag (OOB) balanced accuracy. Model generalization was evaluated using Leave-One-Out Cross Validation (LOOCV), and final performance was assessed on the aggregated LOOCV predictions.

Two independent modeling analyses were performed: one based on the Exercise + Cumulative feature configuration (16:00–17:00 and 16:00–22:00) and one based on the Exercise + Post-exercise feature configuration (16:00–17:00 and 17:00–22:00). The complete modeling workflow adopted for both analyses is summarized in Figure 2. The entire modeling pipeline described below was applied separately to both analyses to ensure methodological consistency and allow direct comparison between the two temporal frameworks.

A Leave-One-Out Cross Validation (LOOCV) was adopted to evaluate generalization performance, which is particularly suitable in small-sample settings as it maximizes the use of available data for training at each iteration [22,23]. In each of the N iterations, the recording session of one subject was held out as the test sample while the N-1 recording sessions of the remaining subjects constituted the training set. All feature selection steps were performed exclusively on the training partition to prevent data leakage.

At each iteration, the training data underwent two feature selection steps: the first step included the removal of zero-variance features, followed by iterative variance inflation factor (VIF) filtering with a threshold of 5, whereby the feature with the highest VIF was iteratively removed until all remaining features satisfied the condition [24]. In the second step, features were ranked using the minimum redundancy–maximum relevance (mRMR) criterion [25], which maximizes mutual information with the target while penalizing inter-feature redundancy; mRMR was preferred over embedded methods for its greater stability on small datasets.

Different top-K configurations (K = 3, 5, and 7) were evaluated to identify the optimal number (K) of mRMR-ranked features and the model complexity that best balanced predictive performance and generalizability. Synthetic Minority Oversampling Technique (SMOTE) [26] with 5 nearest neighbors was subsequently applied on the top-K training features to address class imbalance, after feature selection to avoid influencing the mRMR ranking. A Random Forest (RF) classifier was selected [19,27] and trained on the SMOTE-augmented data, with hyperparameters tuned via grid search and model selection based on Out-Of-Bag (OOB) balanced accuracy. Specifically, within each LOOCV fold, hyperparameter optimization was performed via grid search on the training partition exclusively, with the held-out test sample remaining inaccessible throughout. The search space included the number of trees (100, 150, 200) and the minimum leaf size (5, 10, 15), with the optimal combination selected based on OOB balanced accuracy. A cost matrix penalizing false negatives twice as heavily as false positives was implemented to reflect the clinical cost of missed hypoglycemic events. To further justify the choice of the RF classifier, a post hoc comparison against Logistic Regression (LR), Support Vector Machine (SVM), and Gradient Boosting (GB) was performed on the best-performing feature configurations, applying the same LOOCV framework. To assess the validity of the SMOTE-generated samples, KDE plots were used to compare the feature distributions of real and synthetic NH instances, verifying that the synthetic samples were consistent with the observed data distribution.

### 2.5. Statistical Analysis

The statistical analysis was performed in MATLAB R2024b (The MathWorks, Inc., Natick, MA, USA). For each selected metric, the data were stratified according to the two target groups. Normality of each group’s distribution was assessed using a graphical test of normal distribution. If both groups met the normality assumption, a two sample Student’s *t*-test was applied; otherwise, the non-parametric Wilcoxon rank-sum test was used [19]. Statistical significance for each metric was assessed by two-sided *p*-values, with values below 0.05 considered as statistically significant.

### 2.6. Model Evaluation and Interpretability

Classification performance was assessed using eight metrics—accuracy, balanced accuracy (BA), sensitivity, specificity, precision, F1-score, the area under the receiver operating characteristic curve (ROC-AUC), and the area under the precision-recall curve (PR-AUC)—used to provide a more informative evaluation of performance on the minority class. Final performance was assessed by aggregating the predictions obtained on each held-out sample across all LOOCV iterations, yielding a single set of out-of-sample predictions over the entire dataset. Additionally, 95% confidence intervals were computed for all performance metrics by bootstrapping the LOOCV predictions. Differences between model configurations were further evaluated through bootstrap-based comparisons of ROC-AUC, PR-AUC, and balanced accuracy, as well as McNemar’s tests on classification outcomes, sensitivity, specificity, and balanced accuracy (Bonferroni-corrected).

Model interpretability was supported through two complementary analyses: stability selection, whereby features selected by mRMR in at least 70% of the LOOCV folds were considered stable, and a post hoc OOB permutation importance analysis, in which RF importance scores were averaged across all folds where each feature was selected. The 70% threshold was selected as an empirically motivated conservative criterion to prevent retained features from being artifacts of specific data partitions. In addition, a SHAP (SHapley Additive exPlanations) analysis was performed aggregating SHAP values across all LOOCV folds, in which each feature was selected to provide a consistent estimate of individual feature contributions to the model output.

### 2.7. Independent Cohort Validation

To evaluate the generalizability of the proposed modeling pipeline, the full analysis framework was independently applied to the OhioT1DM 2020 dataset, comprising 6 subjects with T1D (2 males, 4 females) monitored under free-living conditions using a Medtronic Enlite CGM sensor at 5-min intervals [28]. To ensure physiological comparability with the DirecNet protocol, only post-lunch exercise sessions with a minimum duration of 30 min were retained, identified by matching self-reported lunch meal entries and subsequent exercise annotations from the XML dataset files. This selection yielded 84 sessions (11 NH, 73 non-NH) from an initial pool of 168 sessions (22 NH, 146 non-NH). In this case, the NH threshold was set to 70 mg/dL, consistent with the current clinical standard. CGM features were extracted using the same iglu-based pipeline, with temporal windows defined in relation to each session’s self-reported exercise onset and offset, mirroring the DirecNet windowing strategy.

Given the small number of NH events (*n* = 11) and repeated sessions per subject, a stratified group 3-fold cross-validation was adopted to prevent data leakage across subjects while preserving class ratios across folds. SMOTE was not applied due to the insufficient number of minority-class samples; instead, dynamic cost-sensitive learning was used, computing a fold-specific cost matrix proportional to the inverse class frequency of each training partition. The classification threshold was optimized by maximizing the G-mean rather than F1-score, to better balance sensitivity and specificity under the more severe class imbalance of this cohort (NH prevalence: 13.1% vs. 37.5% in DirecNet) [29]. The same two temporal configurations (Exercise + Cumulative top-K = 3 and Exercise + Post top-K = 5) and the full feature selection pipeline were applied independently to this cohort.

## 3. Results

The final dataset consisted of 49 subjects, including 31 subjects without NH and 18 with NH: one subject (ID 35) was excluded due to the absence of nocturnal CGM measurements, precluding the determination of NH. The baseline characteristics for the NH and No-NH groups are reported in Table 1.

VIF-based multicollinearity filtering retained 10 and 12 features for the EX + POST and EX + CUM configurations respectively (Supplementary Tables S4 and S5), with all retained features showing VIF well below the threshold of 5 (mean VIF: 2.33 and 2.89 respectively) and the feature set being invariant across top-K configurations (K = 3, 5, 7), confirming that multicollinearity was effectively resolved prior to mRMR-based feature selection and that the retained features carried mutually independent predictive information.

Across all evaluated top-K configurations, the Exercise + Post framework consistently outperformed Exercise + Cumulative in terms of balanced accuracy, ROC-AUC, and PR-AUC, with top-K = 5 representing the optimal configuration. For the Exercise + Cumulative framework, top-K = 3 yielded the best performance. The complete results across all configurations are reported in Supplementary Table S3 and Figure S1. The best results for the two temporal feature configurations (Exercise + Cumulative and Exercise + Post-exercise) are provided in the following subsections.

### 3.1. Exercise + Cumulative Features Window

#### 3.1.1. Statistical Differences Across Exercise + Cumulative Features

Non-parametric comparisons using the Wilcoxon rank-sum test revealed statistically significant differences for two features: CONGA-15_EX (*p* = 0.030) and MAG (*p* = 0.044). No other features showed statistically significant differences (see Table S2 in the Supplementary Materials).

#### 3.1.2. Exercise + Cumulative Model Performance

**Figure 3:**
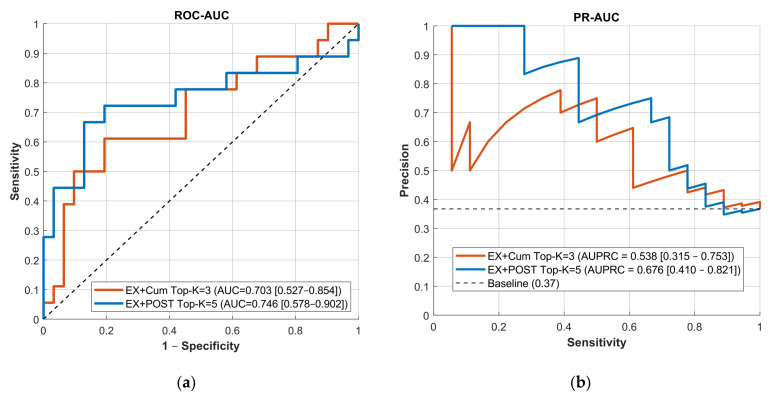
Performance comparison between the Exercise + Cumulative with top-K = 3 (EX + Cum) and Exercise + Post-exercise top-K = 5 (EX + POST) configurations. (**a**) Receiver operating characteristic (ROC) curves, with the dashed line representing a random chance. (**b**) Precision-recall (PR) curves, with the dashed line indicating the baseline prevalence (0.37).

The performance results of the best model are summarized in Table 2. For top-K = 3, the model achieved the best performance in terms of balanced accuracy and F1-score, with a good trade-off between sensitivity and specificity (accuracy = 73.5%, BA = 70.9%, sensitivity = 61.1%, specificity = 80.6%, precision = 64.7%, and F1 = 0.63). The ROC-AUC was 0.70, and the PR-AUC was 0.54 (Figure 3a,b). The related confusion matrix is reported in Figure S6. The performance results obtained by other classifiers are reported in Supplementary Table S8. A comparison of the results obtained with and without SMOTE are reported in Supplementary Table S6.

#### 3.1.3. Feature Stability and Post Hoc Importance for Exercise + Cumulative Configuration

**Figure 4:**
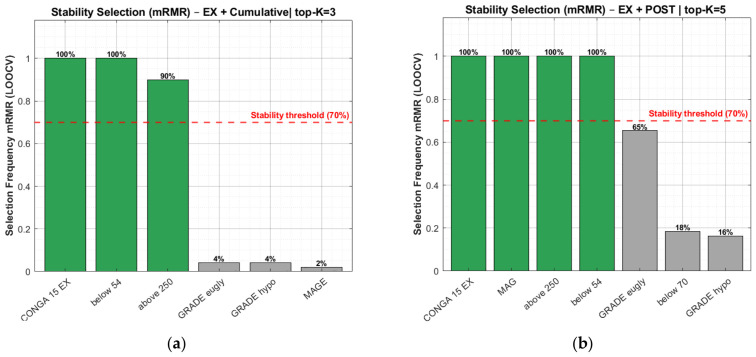
mRMR feature stability selection frequency across LOOCV folds (*n* = 49) for (**a**) Exercise (EX) + Cumulative features, top-K = 3, and (**b**) EX + Post-exercise features, top-K = 5. The dashed line marks the 0.70 stability threshold. Only features above the threshold were considered stable.

**Figure 5:**
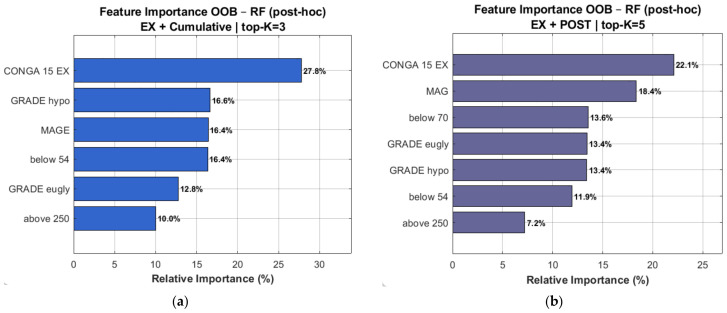
Post hoc OOB Random Forest feature importance (%) for (**a**) Exercise (EX)+ Cumulative features, top-K = 3, and (**b**) EX + Post-exercise features, top-K = 5. Importances are provided for interpretative purposes only and were not used for feature selection.

Stability selection across all LOOCV folds (frequency threshold ≥ 70%) identified three consistently selected features: CONGA-15_EX (100%), below_54 (100%), and above_250 (89.8%) (Figure 4a). Post hoc OOB Random Forest importance attributed the highest predictive weight to CONGA-15_EX (27.8%), followed by GRADE_hypo (16.6%), MAGE (16.4%), below_54 (16.4%), GRADE_eugly (12.8%), and above_250 (10.0%) (Figure 5a). SHAP analysis confirmed the dominant contribution of CONGA-15_EX to individual predictions, while below_54 and above_250 provided complementary directional contributions; the full results are reported in Supplementary Figure S8. KDE plots of the minority class feature distributions before and after SMOTE-based oversampling, for the Exercise + Cumulative best-performing configuration (top-K = 3), are reported in Figure S4 in the Supplementary Materials.

### 3.2. Exercise + Post-Exercise Window

#### 3.2.1. Statistical Differences Across Exercise + Post-Exercise Features

The same statistical testing procedure yielded again the previously reported significant features: CONGA-15_EX (Wilcoxon, *p* = 0.030) and MAG (Wilcoxon, *p* = 0.048). The complete results are reported in Table S2 in the Supplementary Materials.

#### 3.2.2. Exercise + Post-Exercise Model Performance

For this feature set, top-K = 5 provided the best LOOCV performance (Table 3). The model showed an accuracy of 79.6%, BA of 76.9%, sensitivity of 66.7%, specificity of 87.1%, precision of 75.0%, and F1-score of 0.71. The ROC-AUC was 0.75, and the PR-AUC was 0.68 (Figure 3a,b). The related confusion matrix is reported in Figure S7. The performance results obtained by other classifiers are reported in Supplementary Table S9. A comparison of the results obtained with and without SMOTE is reported in Supplementary Table S7.

#### 3.2.3. Feature Stability and Post Hoc Importance for Exercise + Post-Exercise

Stability selection across all LOOCV folds (frequency threshold ≥ 70%) identified four selected features with a selection frequency of 100%: CONGA-15_EX, MAG, above_250, and below_54 (Figure 4b). Post hoc OOB importance ranked CONGA-15_EX first (22.1%), followed by MAG (18.4%), below_70 (13.6%), GRADE_eugly (13.4%), GRADE_hypo (13.4%), below_54 (11.9%), and above_250 (7.2%) (Figure 5b). For the SHAP analysis, CONGA-15_EX again emerged as the leading contributor, with MAG, below_54, GRADE_hypo, and above_250 providing additional complementary contributions in descending order of magnitude; the full results are reported in Supplementary Figure S9. KDE plots of the minority class feature distributions before and after SMOTE-based oversampling, for the Exercise + Post best-performing configuration (top-K = 5), are reported in Figure S5 in the Supplementary Materials.

### 3.3. Summary Results

In summary, CONGA-15_EX emerged as the most robust predictor, being selected in 100% of LOOCV folds and attaining the highest post hoc importance in both configurations (27.8% and 22.1%, respectively). In addition, features characterizing hypoglycemic and hyperglycemic exposure—including below_54, below_70, and above_250—as well as variability descriptors such as MAG (capturing the speed of glucose fluctuations), MAGE (capturing the magnitude of glucose swings), and GRADE_hypo were consistently selected.

No statistically significant differences were found between the two configurations; bootstrap-based comparisons yielded *p*-values of 0.728, 0.502, and 0.528 for ROC-AUC, PR-AUC and balanced accuracy respectively, and McNemar’s tests confirmed no significant differences in accuracy, sensitivity, specificity, or balanced accuracy (all *p* > 0.50).

Additional results for all evaluated top-K configurations, including performance metrics, feature stability, and post hoc importance analyses, are reported in the Supplementary Materials in Table S3 and Figure S1, Figure S2, and Figure S3, respectively.

### 3.4. Independent Cohort Validation Results

**Figure 6:**
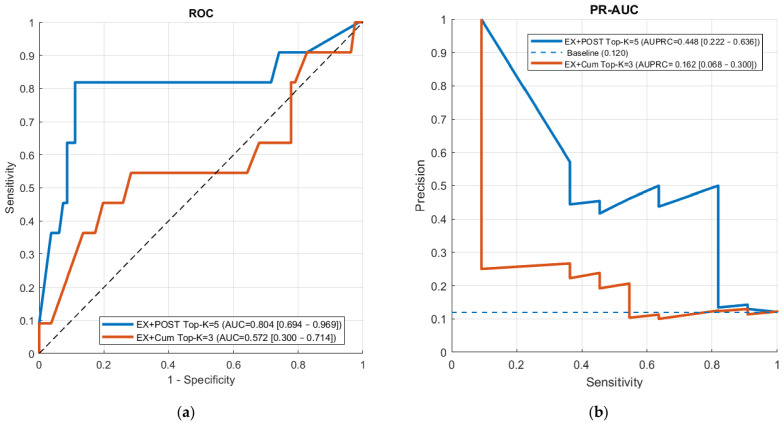
A performance comparison between the Exercise + Cumulative with top-K = 3 (EX + Cum) and Exercise + Post-exercise top-K = 5 (EX + POST) configurations for the independent cohort (OhioT1DM 2020 dataset). (**a**) Receiver operating characteristic (ROC) curves, with the dashed line representing a random chance. (**b**) Precision-recall (PR) curves, with the dashed line indicating the baseline prevalence (0.12).

The performance metrics for both configurations are reported in Table 4, and the corresponding ROC and precision-recall curves are shown in Figure 6. The Exercise + Post configuration achieved balanced accuracy and ROC-AUC consistent with the DirecNet internal results, with a PR-AUC substantially exceeding the no-skill baseline of 0.120, reflecting meaningful discriminative performance despite the more severe class imbalance of the external cohort. The EX + CUM configuration showed markedly lower balanced accuracy and a ROC-AUC approaching chance level, with PR-AUC only marginally exceeding the no-skill baseline.

Regarding feature overlap, two features were shared with the DirecNet selection in both configurations: below_54 appeared in both Exercise + Post and Exercise + Cumulative, and above_250 in Exercise + Post. GRADE_hypo, previously identified as a relevant variability descriptor in DirecNet, was also selected in the Exercise + Post configuration for the independent cohort. The remaining selected features differed: GRADE_hypo, Mean_Glucose_EX, and SDdm were selected in Exercise + Post in place of MAG and below_70, while SDdm replaced CONGA-15_EX as the leading feature in Exercise + Cumulative alongside below_54.

Given the small number of subjects (*n* = 6) and the grouped cross-validation structure, formal statistical comparison between configurations was not performed; differences are reported descriptively and uncertainty is reflected in the fold-level confidence intervals. The wide intervals, particularly for sensitivity, reflect the limited number of NH events (*n* = 11) and should be interpreted with caution.

## 4. Discussion

### 4.1. Novelty and Relevance of the Methodological Approach

This study proposed a methodological approach providing an interpretable early signature based on CGM-derived features to predict the risk of NH following afternoon exercise in a pediatric T1D cohort. The clinical significance of this problem is underscored by the fact that NH in children and adolescents is often asymptomatic, difficult to detect, and associated with severe adverse outcomes including seizures and neurocognitive impairment, yet current CGM-based decision support systems lack dedicated tools for anticipatory NH risk stratification in the exercise context.

The approach has three aspects of novelty. First, the method was designed considering for the first time different temporal CGM segments for the prediction, which were demonstrated to carry different relevant information. Indeed, the Exercise + Post-exercise configuration, which explicitly separates the exercise phase from the subsequent recovery period, emerged as the best-performing approach compared to the cumulative configuration (test set BA: 76.9% vs. 70.9%). This was accompanied by a higher PR-AUC (0.68 vs. 0.54), highlighting improved detection of NH events in a moderately imbalanced setting. Crucially, this directional superiority was independently replicated on the OhioT1DM free-living cohort, where the Exercise + Post-exercise configuration again outperformed the Exercise + Cumulative one across all key performance metrics, providing external corroboration that the temporal separation of exercise and recovery phases captures genuinely more informative glycemic dynamics for NH prediction. The second methodological contribution consists of the adoption of SMOTE-based class balancing within training folds, applied exclusively to the training partition to prevent majority-class bias in the Random Forest classifier without influencing the evaluation of generalization performance. The gap between training BA (89.6%) and test BA (76.9%) is consistent with the expected effect of SMOTE augmentation on training data and does not reflect overfitting of the underlying model, as test performance was evaluated solely on real, non-augmented held-out samples. Finally, the method relies on a temporally structured feature engineering approach. Digital biomarkers are derived from CGM signals by applying established CGM metrics over physiologically relevant time windows, with tuning of metric-specific parameters within the computation functions (e.g., lag in CONGA set to 15 min) to better capture exercise-induced glycemic dynamics.

### 4.2. Physiologic Interpretation of the Digital Signature

Among all the extracted features, CONGA-15_EX emerged as the most robust predictor. As a short-term variability metric computed during the exercise window, CONGA-15_EX captures rapid intra-exercise glucose fluctuations, which may reflect the hepatic glycogen depletion and the progressive increase in insulin sensitivity that characterize moderate-intensity aerobic activity, both of which are known contributors to delayed NH [30]. In addition, MAG emerged as a stable predictor in the Exercise + Post-exercise configuration, where the recovery window was modeled separately, suggesting that aggregating exercise and recovery periods attenuates relevant post-exercise signals. Similarly, GRADE_hypo provided a compact representation of cumulative hypoglycemic burden during the recovery observation period. Overall, these findings support the interpretation that NH risk is not instantaneous but temporally distributed across both exercise and recovery phases. This temporal decomposition represents a conceptual departure from existing approaches, which either extract features from fixed pre-exercise windows or aggregate exercise and post-exercise dynamics into a single representation, potentially obscuring the distinct glycemic signatures of each phase.

### 4.3. Comparison with Existing Approaches

CGM has substantially improved the characterization of NH in T1D, but current clinical decision-support systems remain largely based on threshold alarms and short-horizon predictive features derived from recent glucose dynamics (e.g., trend arrows) [31]. While effective for detecting imminent hypoglycemia, these approaches are not designed to capture delayed glycemic consequences of earlier physiological stressors, such as exercise.

Within the broader literature, multimodal approaches integrating CGM with wearable-derived activity signals have shown promising results, but their clinical applicability is limited by additional sensing requirements and variability in data acquisition protocols [5,14,15]. Conversely, CGM-only predictive models have largely focused on short-term horizons or general night-time hypoglycemia risk, and have reported strong overall performance but only moderate sensitivity to hypoglycemic events (e.g., 55.3%) [12]; furthermore, none of the reviewed CGM-only approaches explicitly models the temporal relationship between exercise and delayed NH onset by separating exercise-phase from recovery-phase glycemic dynamics, which is the defining methodological contribution of the present work. While the overall classification performance in our study remains moderate, the observed sensitivity–specificity trade-off (sensitivity = 66.7%; specificity = 87.1%) suggests that explicitly preserving the temporal separation between exercise and recovery phases may better capture post-exercise glycemic dynamics, improving the identification of hypoglycemic events while maintaining high specificity.

### 4.4. Assessment of the Methodological Approach on Independent Cohort Validation

The independent cohort validation supports the generalizability of the proposed temporal framework rather than of a fixed feature set. The superiority of Exercise + Post was consistent across both cohorts, supporting the conclusion that explicit temporal separation of exercise and recovery phases yields more transferable glycemic signatures. Regarding feature overlap, below_54 was consistently selected across both configurations and both cohorts, confirming its role as a stable predictor of NH risk. Additionally, above_250 was retained in Exercise + Post in both cohorts, while GRADE_hypo, previously identified as a relevant variability descriptor in DirecNet, was also selected in the Exercise + Post Ohio configuration. The main differences concerned the exercise-phase features: CONGA-15_EX, the most robust predictor in DirecNet, was not selected in either Ohio configuration, replaced by SDdm, while MAG and below_70 were replaced by Mean_Glucose_EX in EX + POST. These shifts likely reflect differences in glycemic profiles and exercise characteristics between the controlled inpatient DirecNet protocol and the free-living post-lunch sessions of the Ohio cohort, rather than instability of the framework in itself.

The absence of CONGA-15_EX from both Ohio configurations is particularly noteworthy and may reflect genuine physiological differences between the two populations: the rapid intra-exercise glucose fluctuations captured by CONGA-15_EX may be especially pronounced in pediatric T1D, where growth hormone variability and unpredictable spontaneous activity amplify exercise-induced glycemic instability, rendering this metric less discriminative in an adult free-living cohort with different glycemic dynamics. Alternatively, this shift may partly reflect differences in exercise protocol and data collection between cohorts. Thus, disentangling age-related physiological specificity from cohort-specific confounding requires dedicated validation in age-matched external datasets and represents an important direction for future work.

The present study was conducted on a standardized late-afternoon exercise protocol, which represents a clinically relevant scenario given the well-established association between afternoon exercise and NH risk. Whether the identified recovery-phase signatures generalize to morning or midday exercise cannot be established from the current data. The independent validation on the OhioT1DM dataset, which included free-living post-lunch exercise sessions in a markedly different context from the DirecNet inpatient protocol, provides preliminary evidence that the temporal framework retains predictive relevance beyond the specific afternoon timing used for model development. Nonetheless, validation across different exercise timings, particularly morning fasted exercise where the counter-regulatory response and delayed hypoglycemia risk profile are physiologically distinct, remains an important direction for future work.

It should be noted that NH in the DirecNet cohort was defined as at least one CGM reading below 60 mg/dL, in accordance with the original inpatient protocol under which all clinical interventions and event recording were based on this threshold. While this ensures consistency with the study design, it differs from the current clinical standard of <70 mg/dL, which may limit direct comparability with the recent literature reporting. The independent validation on the OhioT1DM cohort, conducted using the standard < 70 mg/dL threshold, mitigates this concern.

### 4.5. Applications, Limitations and Future Work

These findings suggest that compact and interpretable CGM-derived feature sets can support CGM-based AI-enabled risk stratification for NH following exercise. From an application perspective, this framework is well suited for integration into AI-driven CGM decision-support systems and next-generation automated insulin delivery (AID) platforms. By leveraging temporally structured glucose information, such systems could move beyond short-horizon alerting toward anticipatory nocturnal risk prediction. This may enable proactive interventions such as personalized basal insulin modulation, adaptive overnight monitoring strategies, or targeted alerts for post-exercise nights. Moreover, although insulin administration and carbohydrate intake did not differ significantly between NH and non-NH groups in the DirecNet cohort, their integration as additional input variables in future models represents a promising direction. Insulin on board and recent carbohydrate intake directly modulate the glycemic response to exercise and the magnitude of post-exercise insulin sensitivity increase, and their inclusion alongside CGM-derived features could refine risk stratification.

Limitations in this study include the small sample size (*n* = 49, DirecNet cohort), standardized exercise protocol that may not fully reflect the variability of spontaneous physical activity in everyday life, and the use of an earlier-generation CGM device (Medtronic MiniMed Inc., Northridge, CA, USA). However, these constraints also provided a controlled setting that allowed clearer isolation of delayed post-exercise glycemic effects. Moreover, the use of a blinded CGM system, while less accurate than current devices, avoided behavioral modifications driven by real-time glucose feedback, thereby reducing potential confounding effects on the observed glycemic responses. Future work should focus on validating these findings in larger, more diverse cohorts under free-living conditions and across different age groups. In addition, integration of the identified biomarkers into real-time CGM-based support systems and AID platforms, to enable proactive NH alerts potentially improving time-in-range (TIR) and reducing severe adverse outcomes, represents an important next step toward clinical translation.

## 5. Conclusions

A small set of interpretable CGM-derived features effectively discriminates pediatric T1D subjects at risk of NH following afternoon exercise. Critically, modeling exercise and recovery as distinct temporal phases provides added predictive value over cumulative representations, highlighting the importance of temporal structure in post-exercise glycemic dynamics, a dimension overlooked by existing approaches that either rely on multimodal sensing or extract features from non-physiologically motivated windows. The generalizability of the proposed temporal framework was further supported by independent validation on the OhioT1DM free-living cohort. These findings support the development of lightweight, CGM-only predictive tools that prioritize during- and post-exercise monitoring for early NH detection and safer exercise management in pediatric T1D.

## Figures and Tables

**Table 1:** The baseline characteristics of the NH and No-NH groups.

**Clinical Characteristics**	**NH**	**No-NH**	***p*** **-Value**
Weight (kg)	61.7± 9.7	64.1 ± 11.5	0.47
Height (cm)	168.3 ± 10.3	167.6 ± 9.7	0.82
BMI (kg/m^2^)	21.7 ± 2.4	22.7 ± 3.2	0.25
HbA1C (%)	7.8 ± 0.8	7.8 ± 0.9	0.88
**Pre-Exercise CGM (11:00–16:00)**	**NH**	**No-NH**	***p*** **-Value**
CV (%)	20.3 ± 10.1	17.8 ± 8.7	0.37
Mean (mg/dL)	153.6 ± 61.8	156.24 ± 54.3	0.88
LBGI (dimensionless)	1.2 ± 2.0	1.1 ± 2.4	0.91
**Interventions**	**NH**	**No-NH**	***p*** **-Value**
Bedtime Insulin (U)	3.8 ± 7.1	2.1 ± 2.5	0.24
Snack Carbs (g)	21.3 ± 11.7	25.4 ± 22.3	0.48
IRoute (Pump/MDI)	12/6	15/16	0.22
Gender (F/M)	8/10	14/17	0.96
Snack Carbs (Yes/No)	18/0	24/7	0.46

Data are reported as mean ± standard deviation. NH: nocturnal hypoglycemia; BMI: body mass index; HbA1C: glycated hemoglobin; CV: coefficient of variation; F: female; M: male; MDI: multiple daily injections.

**Table 2:** The LOOCV performance metrics for the Exercise + Cumulative features configuration, top-K = 3.

Performance Metrics	Train (SMOTE)	Test (LOOCV)
Accuracy (%)	81.4 [80.0–82.7]	73.5 [61.2–85.7]
BA (%)	81.4 [80.0–82.7]	70.9 [58.1–83.8]
Sensitivity (%)	86.5 [85.1–87.9]	61.1 [37.5–83.3]
Specificity (%)	76.2 [73.9–78.7]	80.6 [66.1–93.2]
Precision (%)	78.9 [77.2–80.8]	64.7 [40.5–87.9]
F1-score	0.823 [0.811–0.835]	0.629 [0.400–0.804]

Results are performance [confidence intervals]. BA: balanced accuracy.

**Table 3:** The LOOCV performance metrics for the Exercise + Post-exercise configuration, top-K = 5. BA: balanced accuracy.

Performance Metrics	Train (SMOTE)	Test (LOOCV)
Accuracy (%)	89.6 [88.2–90.9]	79.6 [67.3–91.8]
BA (%)	89.6 [88.2–90.9]	76.9 [64.0–89.6]
Sensitivity (%)	92.5 [91.0–93.9]	66.7 [43.8–88.2]
Specificity (%)	86.8 [84.6–88.6]	87.1 [73.0–97.3]
Precision (%)	87.8 [86.1–89.3]	75.0 [52.8–95.2]
F1-score	0.899 [0.887–0.912]	0.706 [0.500–0.875]

Results are performance [confidence intervals]. BA: balanced accuracy.

**Table 4:** The performance metrics for the Exercise + Cumulative and Exercise + Post feature configurations, top-K = 3 and top-K = 5, respectively. BA: balanced accuracy.

Performance Metrics	EX + Cum	Ex + POST
Accuracy (%)	78.3 [67.7–88.1]	85.9 [82.7–91.7]
BA (%)	56.2 [45.0–58.6]	76.3 [55.5–94.7]
Sensitivity (%)	27.3 [0.0–36.4]	63.6 [20.0–100.0]
Specificity (%)	85.2 [71.6–98.1]	88.9 [83.7–94.7]
Precision (%)	43.8 [23.1–53.8]	20.0% [0.0–66.7]
F1-score	0.231 [0.000–0.286]	0.519 [0.209–0.700]

Results are performance [confidence intervals]. BA: balanced accuracy.

## Data Availability

The data used in this study belong to the DirecNet public data repository (Jaeb Center for Health Research, Tampa, FL, USA) freely available at: https://public.jaeb.org/direcnet/stdy/160 [accessed 9 June 2026].

## Supplementary Materials

The following supporting information can be downloaded at https://www.mdpi.com/article/10.3390/s26123842/s1, Figure S1: Performance curves for all top-K configurations across both temporal frameworks, evaluated under LOOCV; Figure S2: Feature selection stability across configurations; Figure S3: Post hoc Random Forest feature importance across configurations; Figure S4: Kernel density estimation (KDE) of the minority class (NH) feature distributions before and after SMOTE-based oversampling, for the Exercise + Cumulative best-performing configuration (top-K = 3); Figure S5: Kernel density estimation (KDE) of the minority class (NH) feature distributions before and after SMOTE-based oversampling, for the Exercise + Post-exercise best-performing configuration (top-K = 5); Figure S6: Confusion matrix for Exercise + Cumulative (top-K = 3) configuration; Figure S7: Confusion matrix for Exercise + Post (top-K = 5) configuration; Figure S8: SHAP plot for Exercise + Cumulative (top-K = 3) configuration; Figure S9: SHAP plot for Exercise + Post (top-K = 5) configuration; Table S1: Complete list of CGM-derived features used in the study; Table S2: Statistical comparison of CGM metrics between the Exercise + Cumulative window and the Exercise + Post-exercise window; Table S3: Classification performance across all evaluated top-K configurations for both temporal analyses (EX + Cumulative and EX + POST); Table S4: Features retained after iterative VIF-based multicollinearity filtering: EX + Post-exercise configuration (representative fold: last LOOCV iteration); Table S5: Features retained after iterative VIF-based multicollinearity filtering: EX + Cumulative configuration (representative fold: last LOOCV iteration); Table S6: Performances obtained with and without SMOTE for the Exercise + Cumulative (top-K = 3) configuration; Table S7: Performances obtained with and without SMOTE for the Exercise + Post (top-K = 3) configuration; Table S8: Performances obtained by other classifiers for the Exercise + Cumulative (top-K = 3) configuration; Table S9: Performances obtained by other classifiers for the Exercise + Post (top-K = 5) configuration.
