# Supplementary material for: Prediction of Nocturnal Hypoglycemia Following Exercise in Type 1 Diabetes Using Temporally Structured CGM-Derived Digital Biomarkers
Source: Sensors (Basel). 2026 Jun 17;26(12):3842. doi: 10.3390/s26123842 (PMC13307307; doi:10.3390/s26123842)
Supplement: Supplementary file 1 [file sensors-26-03842-s001.zip › sensors-4334210-supplementary.pdf]

Supplementary Material

This section provides detailed results for all additional model configurations evaluated in this study. The analyses follow the same LOOCV-based modeling pipeline described in the main manuscript, including feature selection via mRMR, VIF-based multicollinearity filtering, SMOTE-based class balancing, and Random Forest classification with OOB-guided hyperparameter tuning. Results are reported for all top-K configuration (K=3,5,7) across both temporal frameworks – Exercise + Cumulative features and Exercise + Post-exercise features – with the exception of the best-performing configuration (EX + Cumulative top-K=3 and EX + POST top-K=5) already discussed in the main text, which are included here solely for completeness of comparison.

Section S1. Complete List and Definition of CGM-Derived Features

Table S1. Complete list of CGM-derived features used in the study. Features are grouped by physiological category; for each metric, a concise definition, unit of measurement, and physiological interpretation are provided.

Features computed on the Cumulative and Post-exercise window				
**Feature Name**	**Category**	**Definition**	**Unit**	**Interpretation**
ADRR	Risk metric	Average Daily Risk Range combining hypo/hyper risks	–	Overall glycemic risk
COGI	Composite index	Continuous Overall Glucose Index	–	Global glucose control
CV	Variability	SD divided by mean glucose	%	Relative variability
eA1C	Glycemic control	Estimated HbA1c derived from mean glucose	%	Long-term control estimate
GMI	Glycemic control	Glucose Management Indicator	%	CGM-based HbA1c estimate
GRADE	Risk metric	Glycemic Risk Assessment Diabetes Equation score	–	Overall glycemic risk
GRADE_eugly	Risk metric	GRADE contribution from euglycemia	–	Normoglycemia component
GRADE_hyper	Risk metric	GRADE contribution from hyperglycemia	–	Hyperglycemia risk
GRADE_hypo	Risk metric	GRADE contribution from hypoglycemia	–	Hypoglycemia risk
HBGI	Risk metric	High Blood Glucose Index	–	Hyperglycemia risk
LBGI	Risk metric	Low Blood Glucose Index	–	Hypoglycemia risk
hyper_index	Risk metric	Index quantifying hyperglycemic exposure	–	Hyperglycemia burden
hypo_index	Risk metric	Index quantifying hypoglycemic exposure	–	Hypoglycemia burden
IGC	Composite index	Index of Glycemic Control	–	Overall glucose regulation
IQR	Variability	Interquartile range (Q3–Q1)	mg/dL	Robust variability
J_index	Risk metric	Function of mean and SD of glucose	–	Combined glycemic control
M_value	Risk metric	Log-based transformation of glucose deviation	–	Deviation from target glucose
MAD	Variability	Mean absolute deviation from mean glucose	mg/dL	Dispersion
MAGE	Variability	Mean amplitude of glycemic excursions	mg/dL	Major glucose swings
above_140	Time in range	% of time >140 mg/dL	%	Mild hyperglycemia
above_180	Time in range	% of time >180 mg/dL	%	Hyperglycemia
above_250	Time in range	% of time >250 mg/dL	%	Severe hyperglycemia
below_54	Time in range	% of time <54 mg/dL	%	Severe hypoglycemia
below_70	Time in range	% of time <70 mg/dL	%	Hypoglycemia
in_range_63_140	Time in range	% of time 63–140 mg/dL	%	Tight range control
in_range_70_180	Time in range	% of time 70–180 mg/dL	%	Standard time in range
range	Variability	Max − Min glucose	mg/dL	Total spread
SD	Variability	Standard deviation of glucose	mg/dL	Overall variability

Features computed on the Cumulative and Post-exercise window				
**Feature Name**	**Category**	**Definition**	**Unit**	**Interpretation**
Min_	Central tendency	Minimum glucose value	mg/dL	Lowest glucose
x1stQu_	Distribution	First quartile (25th percentile)	mg/dL	Lower distribution bound
Median	Central tendency	Median glucose value	mg/dL	Central value
Mean	Central tendency	Mean glucose value	mg/dL	Average level
x3rdQu_	Distribution	Third quartile (75th percentile)	mg/dL	Upper distribution bound
Max_	Central tendency	Maximum glucose value	mg/dL	Peak glucose
GVP	Variability	Glycemic Variability Percentage (path length-based)	%	Complexity of fluctuations
SD_Roc	Rate of change	SD of glucose rate of change	mg/dL/min	Variability of trends
CV_Measures_Mean	Variability	Mean CV across segments	%	Average relative variability
AUC	Exposure	Area under glucose curve	mg·min/dL	Total glycemic exposure
MAG	Variability	Mean absolute glucose rate of change	mg/dL/min	Instability
SDw	Variability	Within-day SD	mg/dL	Intraday variability
SDhhmm	Variability	Time-of-day SD	mg/dL	Temporal variability
SDwsh	Variability	Within-subject SD (shifted)	mg/dL	Adjusted variability
conga1	Variability	SD of 1-hour lag differences	mg/dL	Short-term variability
GRI	Risk metric	Glycemia Risk Index	–	Combined risk score
**Features computed on the Exercise window**				
**Feature Name**	**Category**	**Definition**	**Unit**	**Interpretation**
Mean_Glucose_EX	Central tendency	Mean glucose during exercise window	mg/dL	Exercise glycemia
SD_Glucose_EX	Variability	SD during exercise window	mg/dL	Exercise variability
CV_percent_EX	Variability	CV during exercise window	%	Relative variability (exercise)
Glucose_Slope_EX	Rate of change	Mean slope during exercise	mg/dL/min	Trend during exercise
CONGA-15_EX	Variability	SD of 15-min lag differences (exercise)	mg/dL	Short-term variability

Section S2. Results of Statistical Analysis

Table S2. Statistical comparison of CGM metrics between the Exercise + Cumulative window and the Exercise + Post-exercise window. Wilcoxon signed-rank test or paired T-Test were applied based on data distribution. Bold values indicate statistical significance ($p < 0.05$).

	Exercise + Cumulative window		Exercise + Post-exercise window	
**Metric**	**Test**	**p-value**	**Test**	**p-value**
**CONGA-15_EX**	**Wilcoxon**	**0.0302**	**Wilcoxon**	**0.0302**
**MAG**	**Wilcoxon**	**0.0442**	**Wilcoxon**	**0.0488**
Min_	Wilcoxon	0.0648	Wilcoxon	0.1125
below_54	Wilcoxon	0.0932	Wilcoxon	0.0563
hypo_index	Wilcoxon	0.2016	Wilcoxon	0.2943
CV_Measures_Mean	T-Test	0.2134	T-Test	0.0634
in_range_70_180	T-Test	0.2160	T-Test	0.2677
CV	T-Test	0.2366	T-Test	0.0736
conga1	T-Test	0.2575	T-Test	0.1494
Median	T-Test	0.3309	Wilcoxon	0.6186
in_range_63_140	T-Test	0.3449	Wilcoxon	0.5337
GRI	T-Test	0.3574	Wilcoxon	0.6625
above_250	Wilcoxon	0.3789	Wilcoxon	0.2330
Mean	T-Test	0.3810	T-Test	0.4095
GMI	T-Test	0.3810	T-Test	0.4095
eA1C	T-Test	0.3810	T-Test	0.4095
AUC	T-Test	0.3872	T-Test	0.4042
GRADE_eugly	Wilcoxon	0.3894	Wilcoxon	0.3725
below_70	Wilcoxon	0.4401	Wilcoxon	0.4141
above_180	Wilcoxon	0.4465	Wilcoxon	0.5683
LBGI	Wilcoxon	0.4612	Wilcoxon	0.5539
GVP	Wilcoxon	0.5136	T-Test	0.7772
CV_percent_EX	T-Test	0.5177	T-Test	0.5177
SDwsh	T-Test	0.5275	T-Test	0.4243
GRADE_hypo	Wilcoxon	0.5335	Wilcoxon	0.5602
M_value	Wilcoxon	0.5969	Wilcoxon	0.8115
MAGE	T-Test	0.6041	Wilcoxon	0.1810
GRADE_hyper	Wilcoxon	0.6209	Wilcoxon	0.4911
x3rdQu_	Wilcoxon	0.6334	T-Test	0.5803
ADRR	T-Test	0.6613	Wilcoxon	0.3781
above_140	Wilcoxon	0.6662	Wilcoxon	0.6186

SD_Roc	T-Test	0.6831	T-Test	0.6887
hyper_index	Wilcoxon	0.6971	Wilcoxon	0.6795
SD	Wilcoxon	0.7167	T-Test	0.7496
Max_	Wilcoxon	0.7795	T-Test	0.7018
GRADE	Wilcoxon	0.7795	Wilcoxon	0.7954
J_index	Wilcoxon	0.7954	Wilcoxon	0.9256
HBGI	Wilcoxon	0.8112	Wilcoxon	0.9668
Glucose_Slope_EX	T-Test	0.8739	T-Test	0.8739
IQR	Wilcoxon	0.9092	Wilcoxon	0.4553
x1stQu_	Wilcoxon	0.9174	Wilcoxon	0.7166
range	T-Test	0.9200	T-Test	0.8769
IGC	Wilcoxon	0.9256	Wilcoxon	0.9587
SD_Glucose_EX	T-Test	0.9375	T-Test	0.9375
SDhhmm	T-Test	0.9552	T-Test	0.7093
SDw	T-Test	0.9552	T-Test	0.7093
COGI	T-Test	0.9649	T-Test	0.8255
MAD	Wilcoxon	0.9669	Wilcoxon	0.7874
Mean_Glucose_EX	Wilcoxon	0.9752	Wilcoxon	0.9752

Section S3. Additional Model Performance Across Configurations

Table S3. Classification performance across all evaluated top-K configurations for both temporal analyses (EX+ Cumulative and EX+POST). Performance was assessed using accuracy, balanced accuracy (BA), sensitivity, specificity, precision, F1-score, area under the receiver operating characteristic curve (ROC-AUC), and area under the precision-recall curve (AUPRC), computed from aggregated LOOCV predictions.

Analysis	Top-K	Acc (%)	BA (%)	Sens (%)	Spec (%)	Prec (%)	F1	ROC-AUC	AUPRC
EX + Cumulative	**3**	**73.5**	**70.9**	**61.1**	**80.6**	**64.7**	**0.63**	**0.70**	**0.54**
	5	69.4	66.5	55.6	77.4	58.8	0.57	0.63	0.55
	7	71.4	71.6	72.2	71.0	59.0	0.65	0.68	0.52
EX + POST	3	75.5	71.3	55.6	87.1	71.4	0.63	0.73	0.67
	**5**	**79.6**	**76.9**	**66.7**	**87.1**	**75.0**	**0.71**	**0.75**	**0.68**
	7	71.4	69.3	61.1	77.4	61.1	0.61	0.70	0.54

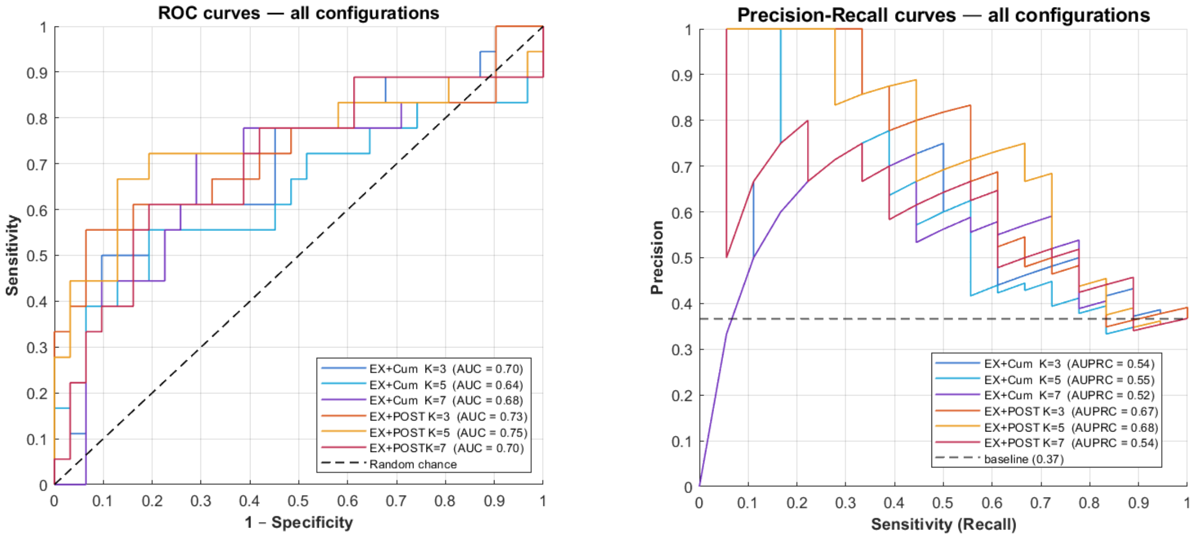

(a) (b)

Figure S1. Performance curves for all top-K configurations across both temporal frameworks, evaluated under LOOCV. **(a)** Receiver Operating Characteristic (ROC) curves; **(b)** Precision-Recall (PR) curves. Results are shown for the EX+ Cumulative configuration (K=3, K=5, K=7; blue-violet palette) and the EX + Post-exercise configuration (K=3, K=5, K=7; orange-red palette). In **(a)**, the diagonal dashed line represents random chance. In **(b)**, the horizontal dashed line indicates the no-skill baseline (prevalence = 0.37).

Section S4. Feature Selection Stability Across Configurations

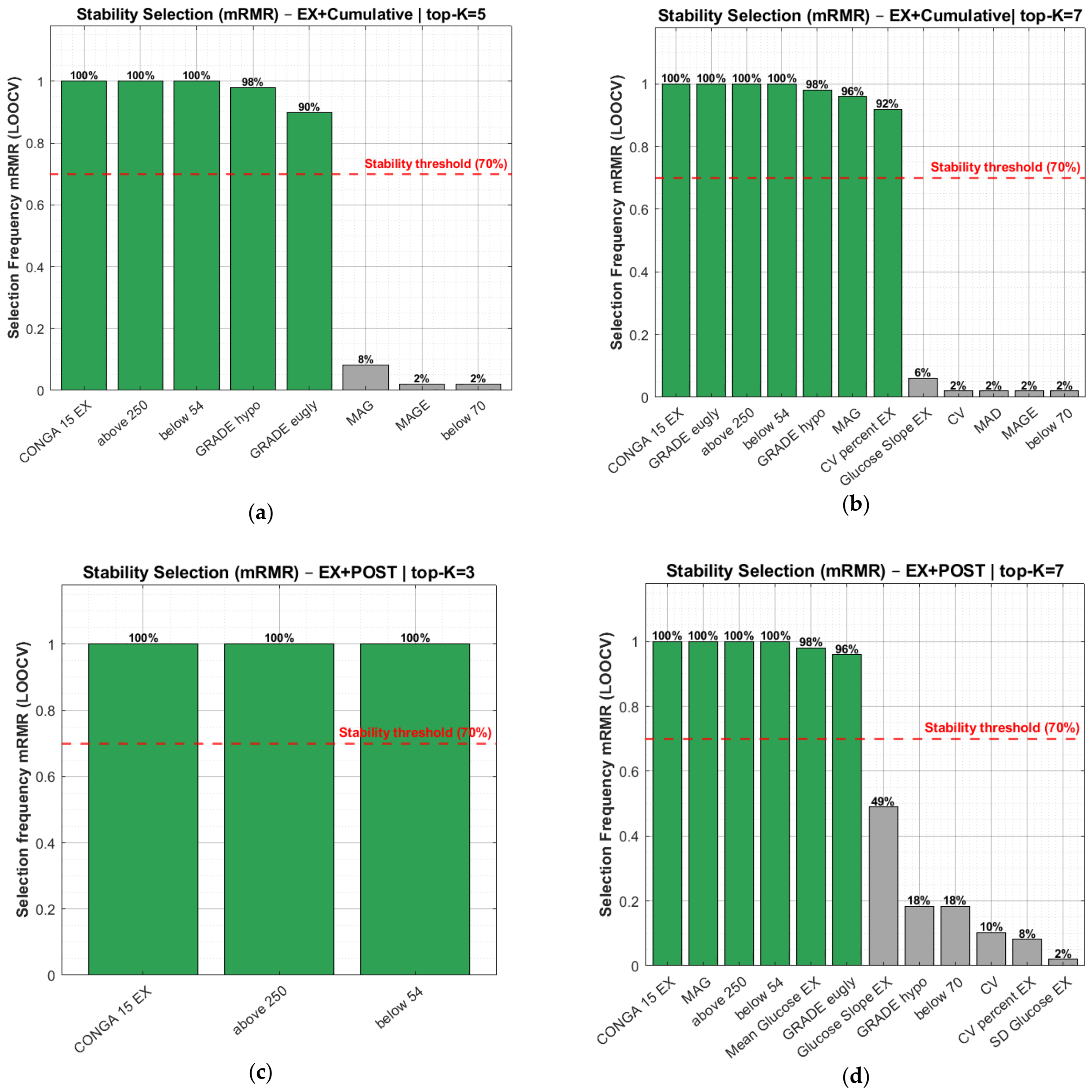

Figure S2. Feature Selection stability across configurations. (**a**) EX+ cumulative, top-K=5; (**b**) EX+ cumulative, top-K=7; (**c**) EX+POST, top-K=3; (**d**) EX+POST, top-K=7.

Section S5. Post-hoc Feature Importance

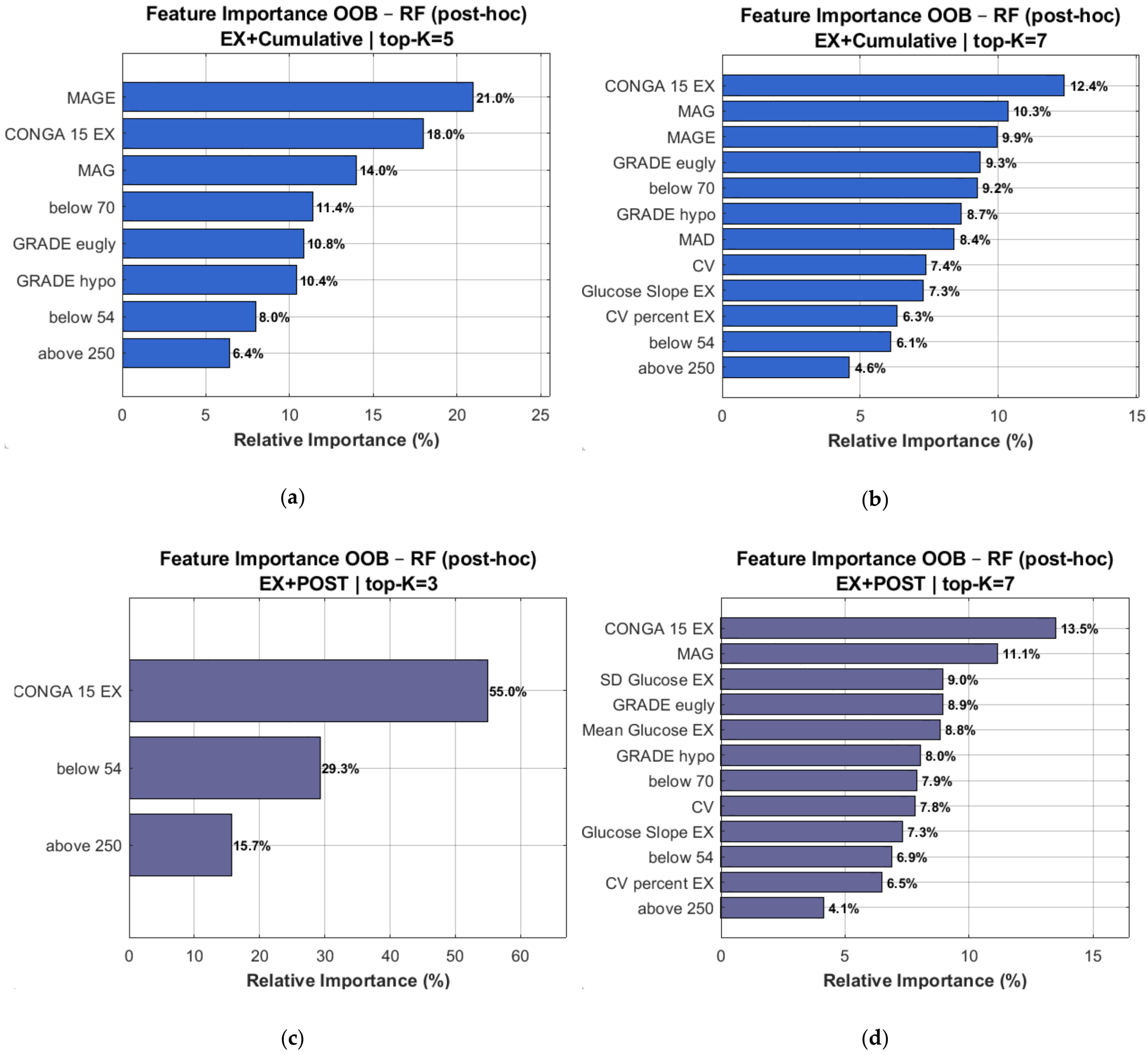

Figure S3. Post-hoc Random Forest feature importance across configurations. Permutation-based feature importance scores were computed using the out-of-bag (OOB) samples of the Random Forest model and averaged across all LOOCV folds in which each feature was selected. Panels show results for: (**a**) Exercise + Cumulative features, top-K=5; (**b**) Exercise + Cumulative features, top-K=7; (c) Exercise + Post-exercise features, top-K=3; (**d**) Exercise + Post-exercise features, top-K=7.

Section S6. Multicollinearity Analysis (VIF)

Table S4. Features retained after iterative VIF-based multicollinearity filtering: EX + Post-exercise configuration (representative fold: last LOOCV iteration). Results are invariant across top-K configurations (K = 3, 5, 7).

Feature	VIF	Tolerance
above_250	1.55	0.65
CV	1.60	0.63
Glucose_slope_EX	1.63	0.62
below_54	1.77	0.56
GRADE_eugly	1.87	0.53
CV_EX	2.50	0.40
GRADE_hypo	2.57	0.39
CONGA-15_EX	2.55	0.39
MAGE	3.41	0.29
MAG	3.88	0.26
**Mean VIF**	**2.33**	

Table S5. Features retained after iterative VIF-based multicollinearity filtering: EX+ Cumulative configuration (representative fold: last LOOCV iteration). Results are invariant across top-K configurations (K = 3, 5, 7).

Feature	VIF	Tolerance
Glucose_slope_EX	1.52	0.66
below_54	1.91	0.53
GRADE_eugly	1.85	0.54
above_250	2.31	0.43
SD_Glucose_EX	2.45	0.41
CONGA-15_EX	2.48	0.40
Mean_Glucose_EX	2.71	0.37
GRADE_hypo	3.23	0.31
MAG	3.56	0.28
GVP	3.91	0.26
MAD	4.01	0.25
CV	4.73	0.21
**Mean VIF**	**2.89**	

Section S7. KDE Distributions (SMOTE validation)

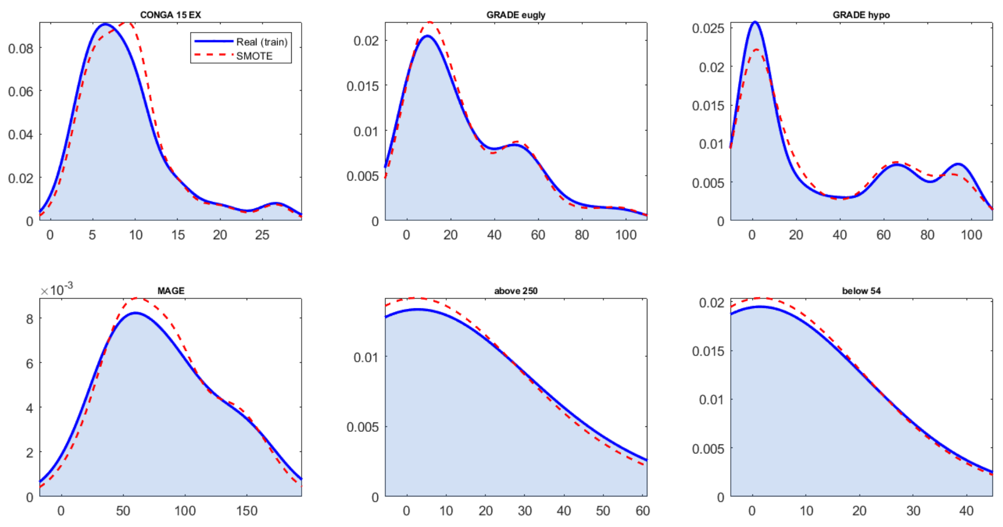

Figure S4. Kernel density estimation (KDE) of the minority class (NH) feature distributions before and after SMOTE-based oversampling, for the Exercise + cumulative best-performing configuration (top-K=3). Each subplot represents the distribution of real training samples (solid blue line, shaded area) with that of SMOTE-generated synthetic samples (dashed red line). Close agreement between the two distributions indicates that synthetic samples were generated within the observed feature space, supporting the validity of the oversampling procedure. The x-axis represents the feature value; the y-axis represents the kernel density estimate.

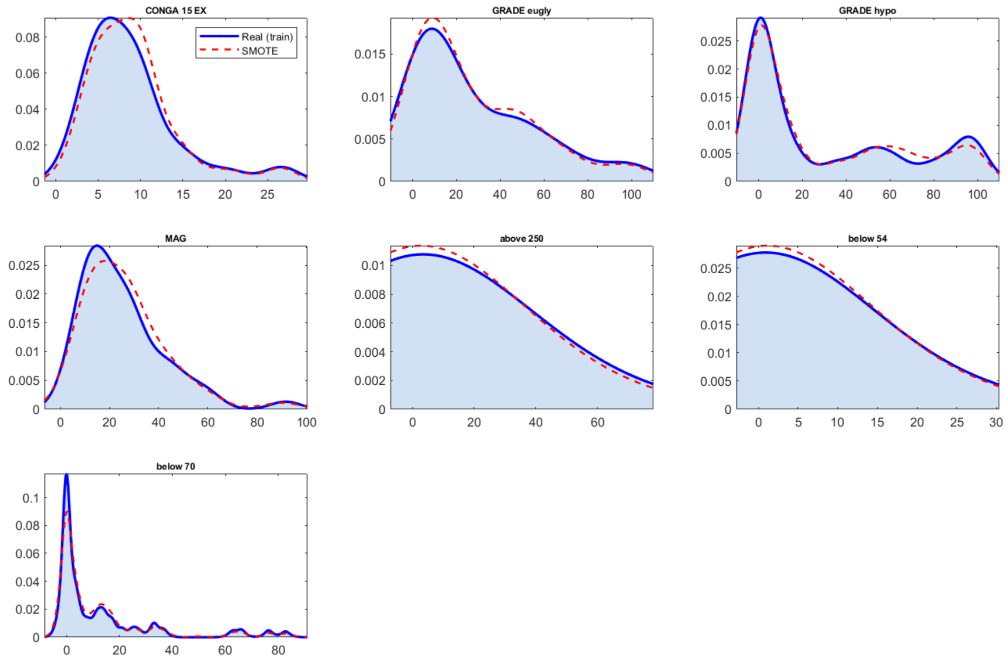

Figure S5. Kernel density estimation (KDE) of the minority class (NH) feature distributions before and after SMOTE-based oversampling, for the Exercise + Post-exercise best-performing configuration (top-K=5). Each subplot represents the distribution of real training samples (solid blue line, shaded area) with that of SMOTE-generated synthetic samples (dashed red line). Close agreement between the two distributions indicates that synthetic samples were generated within the observed feature space, supporting the validity of the oversampling procedure. The x-axis represents the feature value; the y-axis represents the kernel density estimate.

Section S8. SMOTE vs No-SMOTE

Table S6. Performances obtained with and without SMOTE for the Exercise + Cumulative (top-K=3) configuration.

Performance Metrics	SMOTE	No-SMOTE
Accuracy (%)	73.5 [61.2 – 85.7]	73.5 [61.2 – 85.7]
Sensitivity (%)	61.1 [37.5 – 83.3]	72.2 [50.0 – 93.3]
Specificity (%)	80.6 [66.1 – 93.2]	74.2 [58.1 – 88.9]
Precision (%)	64.7 [40.5 – 87.9]	61.9 [40.0 – 83.0]
F1-Score	0.6286 [0.4000 – 0.8038]	0.6667 [0.4800 – 0.8318]
BA (%)	70.9 [58.1 – 83.8]	73.2 [59.5 – 86.4]
AUC	0.7025 [0.5300 – 0.8583]	0.6810 [0.5116 – 0.8460]
AUPRC	0.5382 [0.3150 – 0.7527]	0.4806 [0.2889 – 0.6685]

Results are Performance [Confidence Intervals].

Table S7. Performances obtained with and without SMOTE for the Exercise + Post (top-K=3) configuration.

Performance Metrics	SMOTE	No-SMOTE
Accuracy (%)	79.6 [67.3 – 91.8]	75.5 [63.3 – 87.8]
Sensitivity (%)	66.7 [43.8 – 88.2]	66.7 [43.8 – 88.2]
Specificity (%)	87.1 [73.0 – 97.3]	80.6 [65.6 – 93.0]
Precision (%)	75.0 [52.8 – 95.2]	66.7 [43.6 – 87.5]
F1-Score	0.7059 [0.5000 – 0.8750]	0.6667 [0.4593 – 0.8293]
BA (%)	76.9 [64.0 – 89.6]	73.7 [60.6 – 86.6]
AUC	0.7455 [0.5746 – 0.9028]	0.7151 [0.5386 – 0.8843]
AUPRC	0.6761 [0.4101 – 0.8213]	0.6268 [0.3700 – 0.7865]

Results are Performance [Confidence Intervals].

Section S9. Other Classifiers

Table S8. Performances obtained by other classifiers for the Exercise + Cumulative (top-K=3) configuration.

Performance Metrics	RF	LG	SVM	GB
Accuracy (%)	73.5	61.2	67.3	55.1
Sensitivity (%)	61.1	66.7	61.1	38.9
Specificity (%)	80.6	58.1	71.0	64.5
Precision (%)	64.7	48.0	55.0	38.9
F1-Score	0.6286	0.5581	0.5789	0.3889
BA (%)	70.9	62.4	66.0	51.7
AUC	0.7025	0.6434	0.6039	0.5484
AUPRC	0.5382	0.4419	0.4595	0.3769

Table S9. Performances obtained by other classifiers for the Exercise + Post (top-K=5) configuration.

Performance Metrics	RF	LG	SVM	GB
Accuracy (%)	79.6	65.3	53.1	63.3
Sensitivity (%)	66.7	83.3	77.8	55.6
Specificity (%)	87.1	54.8	38.7	66.7
Precision (%)	75.0	51.7	42.4	50.0
F1-Score	0.7059	0.6383	0.5490	0.5263
BA (%)	76.9	69.1	58.2	61.6
AUC	0.7455	0.6631	0.6434	0.6971
AUPRC	0.6761	0.4366	0.6028	0.5506

Section S10. Confusion Matrices of the best configurations

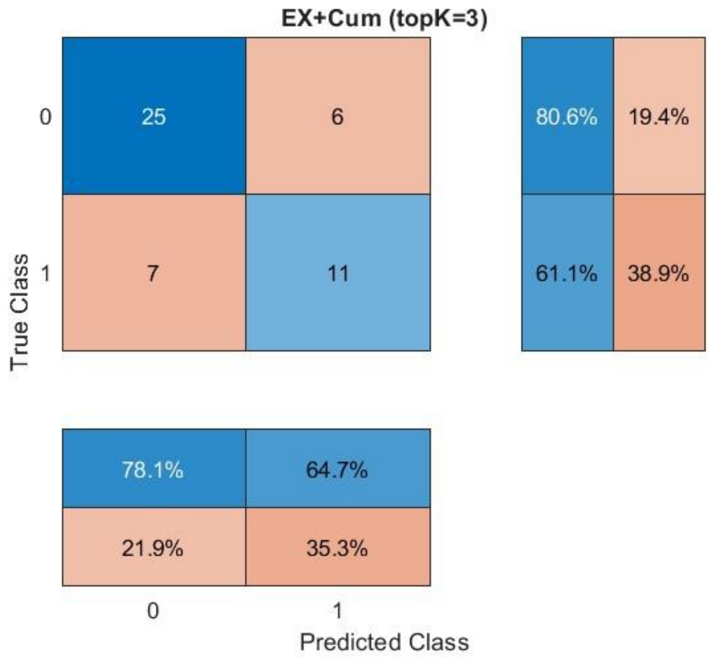

Figure S6. Confusion matrix for Exercise + Cumulative (top-K=3) configuration.

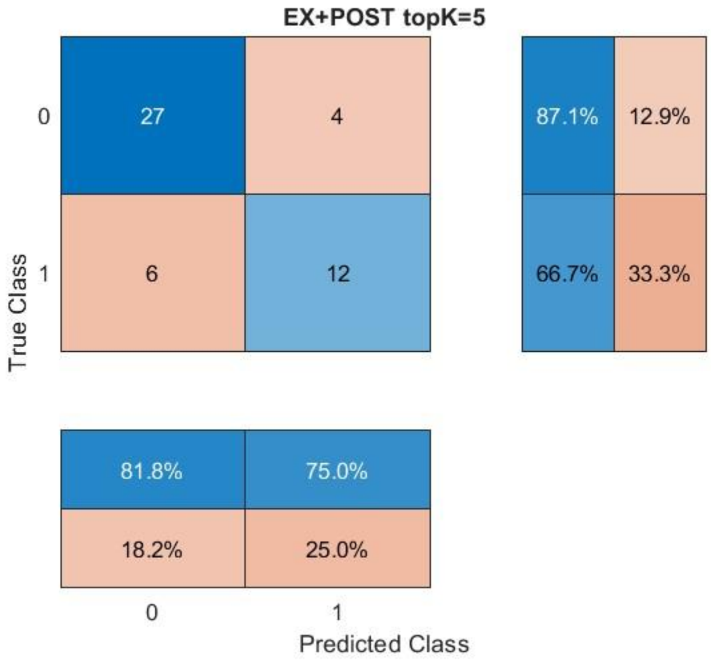

Figure S7. Confusion matrix for Exercise + Post (top-K=5) configuration.

Section S11. SHAP Analysis

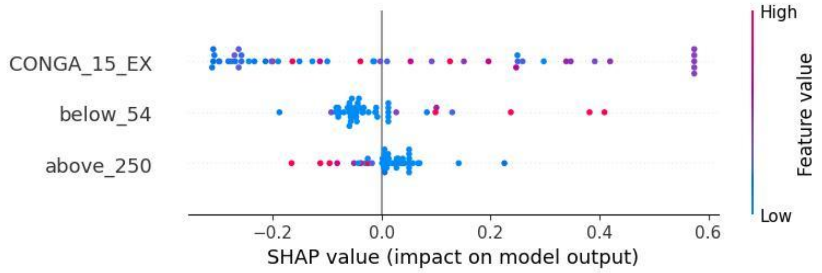

Figure S8. SHAP plot for Exercise + Cumulative (top-K=3) configuration.

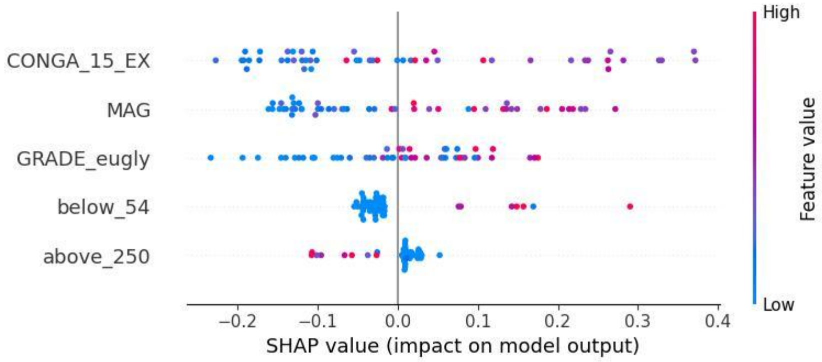

Figure S9. SHAP plot for Exercise + Post (top-K=5) configuration.
